# Nonalcoholic Fatty Liver Disease in Canadian First Nations and Non-First Nations Patients

**DOI:** 10.1155/2016/6420408

**Published:** 2016-04-28

**Authors:** Julia Uhanova, Gerald Minuk, Federico Lopez Ficher, Natasha Chandok

**Affiliations:** ^1^Section of Hepatology, Department of Internal Medicine, University of Manitoba, 804D-715 McDermot Avenue, Winnipeg, MB, Canada R3E 3P4; ^2^Division of Gastroenterology, University of Western Ontario, Room ALL-107, 339 Windermere Road, London, ON, Canada N6A 5A5

## Abstract

*Background.* Features of nonalcoholic fatty liver disease (NAFLD) have yet to be described in the Canadian First Nations (FN) population. The aim of this study was to compare the prevalence, severity, and outcome of NAFLD in FN versus non-FN patients at an urban, tertiary care centre.* Methods.* Adults with NAFLD and no additional liver disease were identified in a prospectively derived database at the University of Manitoba. Demographic, clinical, laboratory, imaging, and histologic data were analyzed.* Results.* 482 subjects fulfilled diagnostic criteria for NAFLD, including 33 (7%) FN. Aside from rural residence, diabetes and cholestasis being more common in FN patients, the ages, gender distributions, clinical and radiologic features, and liver enzyme/function test results were similar in the two cohorts. Noninvasive tests of fibrosis (APRI and NAFLD fibrosis scores) were also similar in the two cohorts. There were no significant differences in liver enzyme or function tests in either cohort after approximately three years of follow-up.* Conclusion.* Compared to the prevalence of FN persons in the general population of this study site (10–15%), FN patients were underrepresented in this NAFLD population. The severity and progression of liver disease in FN patients appear to be similar to those in non-FN patients.

## 1. Introduction

Diabetes and obesity are well established risk factors for nonalcoholic fatty liver disease (NAFLD) [1]. Thus, as the prevalence of diabetes and obesity continues to increase in developed nations, there has been an accompanying increase in the prevalence of NAFLD such that the estimated prevalence of NAFLD in North America is approaching 30% of the adult population [2–4]. Diabetes and obesity are particularly common in the Canadian First Nations (FN) population where prevalence rates are 2–5 times higher than in non-FN populations [5–10]. However, the prevalence and features of NAFLD in Canadian FN patients have yet to be described and compared to non-FN NAFLD patients.

Nonalcoholic steatohepatitis (NASH) is the progressive form of NAFLD that can evolve into cirrhosis and liver failure. NASH is most common in NAFLD patients with metabolic syndrome (MS), a syndrome that can be defined by the presence of three or more of the following comorbid conditions: obesity, diabetes, hyperlipidemia, and hypertension [11–20]. NASH patients also tend to have elevated serum aminotransferase values [21–23]. Approximately 20% of NAFLD patients have NASH [24–29]. Once again, the prevalence and features of NASH in the Canadian FN population have yet to be described.

The factor(s) responsible for the development of NASH and its progression to cirrhosis and liver failure remain unidentified. Recently, data have accrued suggesting that a heightened innate immune response of the liver to enteric derived mitogens plays an important role in the natural history of NAFLD/NASH [30–34]. Given that innate immune responsiveness varies between different ethnicities and amongst various socioeconomic groups [35–41], it seems reasonable to propose that the course of NASH in FN patients might differ from that of non-FN patients.

The purpose of the present study was to determine whether the prevalence, severity, and natural history of NAFLD and NASH differ in FN compared to non-FN NAFLD patients.

## 2. Methods

### 2.1. Patient Population

All patients aged ≥ 18 years with a diagnosis of NAFLD were identified from a prospectively derived patient database maintained by the Section of Hepatology at the University of Manitoba, the principal referral centre for liver disease in the province. The diagnosis of NAFLD had been established on the basis of liver imaging or a liver biopsy compatible with fatty liver; the presence of ≥1 feature of the MS, with or without abnormal liver enzymes; and alcohol consumption ≤ 20 g/day. Patients were excluded from analysis if they had evidence of a concurrent overlapping liver disease. Patients were identified as FN based on chart documentation and/or treaty card number.

### 2.2. Data Collection

A review of patient charts and electronic medical records was undertaken to collect all relevant demographic, clinical, laboratory, histology, and imaging data. Both baseline and follow-up liver biochemistry data were also recorded. The study variables included age at presentation, gender, urban versus rural residence, weight, height, body mass index (BMI), selected comorbidities (diabetes, hyperlipidemia, and hypertension), clinical or radiologic signs of advanced liver disease (e.g., hepatomegaly, splenomegaly, ascites, esophageal varices, and hepatic encephalopathy), complete blood counts, serum alanine (ALT) and aspartate (AST) aminotransferases, alkaline phosphatase, gamma glutamyltransferase (GGT), total bilirubin, total cholesterol, triglycerides, albumin, creatinine, international normalized ratio (INR) for prothrombin times, and glucose. Liver biopsy findings were described as the presence or absence of NASH according to the NAFLD activity score (NAS) with a NAS ≥5 being considered diagnostic by a liver pathologist unaware of the patient's ethnicity. The extent of lobular inflammatory activity was graded on a scale of 0–4 with 0 being no inflammatory activity and 4 panlobular inflammation. Staging of fibrosis was also graded on a scale of 0–4 with 0 representing no increased fibrosis and 4 cirrhosis. In addition, the extent of fibrosis was estimated noninvasively by APRI calculations and the NAFLD fibrosis score as described by Angulo et al. [42].

### 2.3. Statistical Analysis

Continuous variables were reported as means and standard deviations, while categorical variables were reported as percentages. Patients were stratified based on FN versus non-FN status. Chi-square test of association was used to examine differences in demographic factors and in frequencies of clinical variables, such as presence or absence of certain comorbidities. A Yates correction was used where expected counts were less than 5. To assess quantitative differences between the groups, two sample *t*-tests were performed. Statistical analysis was performed using SAS Version 9.1 (SAS Inc., Cary, NC). For all analyses, statistical significance was set at less than 5%.

## 3. Results

Of the 482 patients diagnosed with NAFLD, 33 (7%) were FN. The demographic, clinical, biochemical, and imaging features of these and the 449 non-FN patients are provided in Table 1. Both cohorts had a mean age of 47 years at diagnosis. The majority of FN (67%) and non-FN (52%) patients were female. Fewer FN patients resided in urban centres (24% versus 51%, *p* = 0.003). Aside from FN patients being more often diagnosed with diabetes (42% versus 15%, *p* = 0.0001) the prevalence rates of obesity/overweight, hyperlipidemia, and hypertension were similar in the two cohorts.

On physical examination, BMIs were similar. FN patients more often had hepatomegaly than non-FN patients (30% versus 12%, *p* = 0.002) but splenomegaly was uncommon in both (3% and 6%, resp.). Although signs of advanced liver disease (ascites, varices, and encephalopathy) were only present in non-FN patients, the differences between the two cohorts were not statistically significant.

The results of laboratory testing are provided in Table 2. Mean serum ALT, AST, and GGT levels were similar in the two cohorts but alkaline phosphatase levels were higher in FN patients (151 ± 77 versus 114 ± 61 IU/L, *p* = 0.0002). In terms of liver function testing, serum albumin levels were significantly lower in FN patients (38 ± 3.9 versus 40 ± 6.1 g/L, *p* = 0.01). Total bilirubin levels were also lower in FN patients (9.2 ± 6.4 versus 13 ± 3.2 umol/L) but the differences did not reach statistical significance (*p* = 0.07). INR values were similar as were platelet, total cholesterol, triglyceride, and creatinine values. Serum glucose levels were higher in FN patients (8.2 ± 4.3 versus 7.0 ± 7.5, *p* = 0.006).

Mean durations of follow-up were similar in the two cohorts (FN: 2.9 ± 3.3 years; non-FN: 2.7 ± 3.1 years). Follow-up laboratory testing revealed no significant differences in liver enzyme or function tests within the two cohorts that were not present at baseline.

The results of liver histology and noninvasive indicators of hepatic fibrosis are provided in Table 3. Ninety-six patients (20%) underwent liver biopsy (11 FN and 85 non-FN). NASH was present in 36% of FN patients and 28% of non-FN patients (*p* = 0.84). The extent of hepatic inflammatory activity as graded on a scale of 0–4 was similar, with all FN patients having grade 0–2 inflammation compared to 90.5% of non-FN patients (*p* = 0.63). The extent of fibrosis was also similar in the two cohorts. Specifically, 100% of FN and 90.5% of non-FN patients had stage 0–2 fibrosis (*p* = 0.63). Cirrhosis was uncommon (<5%) in both cohorts. According to the noninvasive APRI and NAFLD fibrosis scores [42], which were applied to all 482 subjects, there were no significant differences in the extent of hepatic fibrosis.

## 4. Discussion

The results of this study suggest that, despite increased prevalence of diabetes and obesity in the Canadian FN population, the prevalence of NAFLD does not appear to be increased in this population. The results also indicate that the severity of liver disease in FN patients is similar to that of non-FN patients with NAFLD. Finally, these preliminary results suggest that NAFLD does not progress at a rate different in FN compared to non-FN NAFLD patients.

Census data estimate that approximately 12% of Manitobans are FN [43], yet only 7% of the NAFLD patients in this tertiary care referral centre were FN [34]. One possible explanation for this would be FN patients being diagnosed more often with additional causes of liver disease such as chronic viral hepatitis, alcohol, or autoimmune liver diseases as has been reported previously [40, 44, 45]. Such patients would have been excluded from analysis in the present study. Also to be considered are differences in access to the health care system with FN patients less often seeking medical care and less often being referred to urban tertiary care centres for further evaluation and/or management. On the other hand, in support of the 7% figure being an overestimate is the fact the health care facility in this study (the Health Sciences Centre) is located in an area of the city where the FN population is highest and the same facility also serves as a primary care centre for this segment of the city's population. Clearly, population-based studies are required to definitively determine whether the prevalence of NAFLD is higher, lower, or similar to that of the non-FN population.

There are a number of means of assessing the severity of liver disease in NAFLD patients. Included amongst these are serum aminotransferase levels and liver histology. In the present study, serum ALT and AST values at baseline were similar in FN and non-FN patients. Histologically, NASH, the aggressive form of NAFLD, was equally present in FN and non-FN patients. Likewise, when inflammatory activity was graded on a scale of 0–4, FN patients had similar activity to non-FN patients.

Because it remains unclear why some NAFLD patients develop active necroinflammatory disease while others do not and what regulates the extent of that activity, the explanation for why FN patients may have less inflammatory disease activity than non-FN patients remains to be determined. However, significant differences in pro- and anti-inflammatory cytokine and Toll-like receptor expression in the innate immune system of FN compared to non-FN patients have been described previously [38, 39].

The follow-up period of approximately three years was likely too short to provide robust insights into the course of NAFLD in FN and non-FN patients. However, surrogate markers of progression such as clinical features of advanced liver disease, biochemical and hematologic evidence of hepatic dysfunction, and the extent of fibrosis on liver biopsy should be considered. In the present study, only non-FN patients had clinical evidence of advanced liver disease including the presence of esophageal varices, ascites, and/or hepatic encephalopathy. In terms of laboratory evidence of advanced disease, although serum albumin levels were lower in FN patients (which could be explained in part by the higher prevalence of diabetes in this cohort), total bilirubin levels trended to being lower and INR values were similar in the two cohorts, suggesting no significant differences in liver function. On liver biopsy, FN patients had less fibrosis than non-FN patients and, according to the noninvasive APRI and NAFLD fibrosis scores, there were no significant differences in fibrosis. Thus, overall, there were no compelling clinical, biochemical, radiologic, or histologic findings to suggest that NAFLD is more or less progressive in FN versus non-FN patients. Once again, long-term, prospective studies involving larger patient cohorts are required to resolve this important question.

This study has a number of strengths and limitations. The main strength is that it represents the first attempt to describe the features of NAFLD in a Canadian FN population. Another strength is the rigorous nature by which patients with concurrent liver diseases were excluded to minimize potential confounding. Additionally, patients had a follow-up period, albeit short, to document significant changes in liver enzyme and function tests. The main limitations to this study were the retrospective, single centre study design, the selection bias inherent in patients being referred to a tertiary care centre, the relatively small number of FN patients identified, the absence of histology in the majority of patients, and, as mentioned previously, the relatively short duration of follow-up.

In conclusion, in this retrospective, single centre, non-population-based study, NAFLD was not more common, severe, or progressive in FN compared to non-FN Canadians but further population-based research in this important area is clearly warranted.

## Figures and Tables

**Table 1:** Demographic and clinical features of First Nations and Non-First Nations patients with nonalcoholic fatty liver disease.

	First Nations		Non-First Nations		
Variable	*N* = 33		*N* = 449		*p*
	*n*	%	*n*	%	
Demographics					
Age at presentation (yrs)^*∗*^	46.6 ± 12.4		46.9 ± 13.1		0.90
Female	22	66.7	233	51.9	0.10
F/M ratio	2		1.1		
Urban	8	24.2	228	50.8	**0.003**
Comorbidities					
Obesity	19	57.6	213	47.4	0.26
Overweight	6	18.2	53	11.8	0.28
Type 2 DM	14	42.4	69	15.4	**0.0001**
Hyperlipidemia	8	24.2	124	27.6	0.67
Hypertension	3	9.1	58	12.9	0.52
Physical findings					
Weight (kg)^*∗*^	97 ± 29		93 ± 19		0.47
BMI^*∗*^	31 ± 5.4		29 ± 6.6		0.10
Hepatomegaly	10	30.3	52	11.6	**0.002**
Splenomegaly	1	3.0	25	5.6	0.53
Ascites	0	0	8	1.8	0.44
Varices	0	0	7	1.6	0.47
Encephalopathy	0	0	5	1.1	0.54
Cirrhosis	1	3.0	19	4.2	0.97
Decompensated cirrhosis	0	0	14	3.1	0.30

^*∗*^Results represent mean ± SD.

yrs: years; F/M: female/male; type 2 DM: type 2 diabetes mellitus.

**Table 2:** Laboratory findings in First Nations and Non-First Nations patients with nonalcoholic fatty liver disease^*∗*^.

Variable (normal range)	First Nations Baseline/follow-up	Non-First Nations Baseline/follow-up	*p* value Baseline/follow-up
Duration of follow-up (yrs)	2.9 ± 3.3	2.7 ± 3.1	0.731
ALT (<30 U/L)	81 ± 52/59 ± 41	83 ± 109/53 ± 43	0.92/0.49
AST (10–32 U/L)	50 ± 31/43 ± 24	58 ± 101/37 ± 22	0.66/0.13
ALP (30–120 U/L)	151 ± 77/136 ± 72	114 ± 61/102 ± 59	**0.0002/0.004**
GGT (5–38 U/L)	119 ± 104/118 ± 119	124 ± 177/110 ± 191	0.88/0.82
Albumin (33–45 G/L)	38 ± 3.9/38 ± 4.1	40 ± 6.1/40 ± 5.1	**0.01/0.02**
Total bilirubin (3–21 *μ*mol/L)	9.2 ± 6.4/8.5 ± 5.0	13 ± 32/12.3 ± 18	0.07/**0.02**
INR (0.9–1.1)	1.0 ± 0.1/1.0 ± 0.1	1.4 ± 1.5/1.1 ± 0.6	0.72/0.57
Platelets (140–440 × 10^9^/L)	279 ± 80/243 ± 83	244 ± 71/239 ± 71	**0.015/**0.78
Creatinine (35–106 *μ*mol/L)	69 ± 21/70 ± 22	77 ± 26/81 ± 46	0.164/0.07
Total cholesterol (<5.2 mmol/L)	5.4 ± 0.9	6.9 ± 17	0.682
Triglycerides (<1.7 mmol/L)	3.0 ± 2.2	5.2 ± 20	0.612
Glucose (3.6–6.0 mmol/L)	8.2 ± 4.3	7.0 ± 7.5	**0.006**

^*∗*^Results represent mean ± SD.

ALT: alanine aminotransferase; AST: aspartate aminotransferase; ALP: alkaline phosphatase; GGT: gamma glutamyltransferase; INR: international normalization ratio.

**Table 3:** Invasive and noninvasive tests for advanced liver disease in First Nations and Non-First Nations patients with nonalcoholic fatty liver disease.

	First Nations		Non-First Nations		
Variable	*N* = 33		*N* = 449		*p*
	*n*	%	*n*	%	
Liver histology					
Liver biopsy performed	11	33.3	85	18.9	**0.045**
NASH	4	36.4	24	28.2	0.84
Grade 0–2 inflammation	11	100	77	90.6	0.63
Grade 3-4 inflammation	0	0	3	3.5	
Stage 0–2 fibrosis	11	100	77	90.6	0.63
Stage 3-4 fibrosis	0	0	8	9.4	
APRI^1^					
APRI at baseline	0.67 ± 0.8		0.75 ± 1.2		0.63
APRI at follow-up	0.51 ± 0.3		0.50 ± 0.4		0.97
NAFLD fibrosis score^2^					0.74
Predictive of F0–F2 fibrosis	20	60.6	299	66.6	
Indeterminate score	10	30.3	112	24.9	
Predictive of F3-F4 fibrosis	3	9.1	38	8.5	

^1^Results represent mean ± SD.

^2^From Angulo et al. [42]: the NAFLD fibrosis score, a noninvasive system that identifies liver fibrosis in patients with NAFLD.

NASH: nonalcoholic steatohepatitis; APRI: AST-to-Platelet Ratio Index; NAFLD: nonalcoholic fatty liver disease.
